# Acidic Stress Induces Cytosolic Free Calcium Oscillation, and an Appropriate Low pH Helps Maintain the Circadian Clock in Arabidopsis

**DOI:** 10.3390/plants13213107

**Published:** 2024-11-04

**Authors:** Wei Chen, Jing Xu, Jia Chen, Jun-Feng Wang, Shu Zhang, Zhen-Ming Pei

**Affiliations:** 1 College of Life Sciences, Zhejiang University, Hangzhou 310027, China; 2 Center on Plant Environmental Sensing, College of Life and Environmental Sciences, Hangzhou Normal University, Hangzhou 310036, China; 3 Institute of Vegetables, Shandong Academy of Agricultural Sciences, Jinan 250100, China; 4 Shandong Key Laboratory of Bulk Open-Field Vegetable Breeding, Ministry of Agriculture and Rural Affairs, Key Laboratory of Huang Huai Protected Horticulture Engineering, Institute of Vegetables, Shandong Academy of Agricultural Sciences, Jinan 250100, China; 5 Department of Biology, Duke University, Durham, NC 27708, USA

**Keywords:** *Arabidopsis thaliana*, calcium imaging, aequorin, calcium signaling, basal cytosolic Ca^2+^ level, acid stress, low pH

## Abstract

Acidic stress is a formidable environmental factor that exerts adverse effects on plant growth and development, ultimately leading to a potential reduction in agricultural productivity. A low pH triggers Ca^2+^ influx across the plasma membrane (PM), eliciting distinct responses under various acidic pH levels. However, the underlying mechanisms by which Arabidopsis plant cells generate stimulus-specific Ca^2+^ signals in response to acidic stress remain largely unexplored. The experimentally induced stimulus may elicit spikes in cytosolic free Ca^2+^ concentration ([Ca^2+^]_i_) spikes or complex [Ca^2+^]_i_ oscillations that persist for 20 min over a long-term of 24 h or even several days within the plant cytosol and chloroplast. This study investigated the increase in [Ca^2+^]_i_ under a gradient of low pH stress ranging from pH 3.0 to 6.0. Notably, the peak of [Ca^2+^]_i_ elevation was lower at pH 4.0 than at pH 3.0 during the initial 8 h, while other pH levels did not significantly increase [Ca^2+^]_i_ compared to low acidic stress conditions. Lanthanum chloride (LaCl_3_) can effectively suppress the influx of [Ca^2+^]_i_ from the apoplastic to the cytoplasm in plants under acid stress, with no discernible difference in intracellular calcium levels observed in Arabidopsis. Following 8 h of acid treatment in the darkness, the intracellular baseline Ca^2+^ levels in Arabidopsis were significantly elevated when exposed to low pH stress. A moderately low pH, specifically 4.0, may function as a spatial-temporal input into the circadian clock system. These findings suggest that acid stimulation can exert a continuous influence on intracellular calcium levels, as well as plant growth and development.

## 1. Introduction

Acid stress is becoming an increasingly severe environmental issue [1,2]. Both the area affected by acid rain and the acidity of rainwater are increasing, and the situation could worsen soon [3]. While increased fertilizer use has increased crop yields worldwide, improper management has caused severe soil acidification, which has had a negative impact on crop yields, especially in China [4]. Acid rain could damage plant cell membrane systems and could negatively affect respiration, photosynthesis, and the antioxidative enzyme system [5,6,7,8]. The decrease in soil pH value will reduce the utilization of soil nutrients (phosphorus, potassium, calcium, and magnesium) and affect the structure and function of microbial communities while increasing the effects of toxic heavy metals (such as cadmium and lead) and other harmful elements (such as aluminum and manganese), especially at a pH below 4.5 [9]. Plants cannot move and are confronted with changing environmental conditions, such as light, water, biotic and abiotic stress. The vacuole regulates osmotic pressure, cytosolic ion homeostasis, protein degradation, and the storage of nutrients and secondary metabolic compounds to cope with harsh environmental conditions. By using a positive charge surplus on the inside of the concentration and the electrostatic gradient, the vacuole can pump other ions and metabolites against their concentration gradient into the vacuole to maintain osmotic balance and functions [10].

The cytosolic pH in *Arabidopsis* is at ~pH 7.4, which was maintained by organic acids and phosphates and plasma membrane-resident proton pumps [11]. In the Arabidopsis, two V-ATPases and one vacuolar pyrophosphatase pump assess protons from the cytosol into the vacuole to establish the proton gradient through ATP energy and pyrophosphate [12]. In the *Arabidopsis* endomembrane system, the pH drops from 7.1 in the endoplasmic reticulum to 5.6 in the trans-Golgi network/early endosome, and vacuoles of *Arabidopsis* root cells typically have a pH of 5.8 [13,14,15]. Changes in the pH of the medium surrounding *Arabidopsis* roots have shown a substantial effect on the apoplastic pH but not cytosolic pH [16]. According to the “acid growth hypothesis”, auxin mediates the SCF^TIR1/AFB^ signaling pathway [17,18] and promotes the expression of SMALL Auxin UP RNA (SAUR) [19], which binds to PM-targeted PP2C.D2/PP2C.D5/PP2C.D6 [19,20,21]. The binding to the PM H^+^ -ATP complex inhibits the dephosphorylation of Thr947, keeping these protons pumping phosphorylation and leading toward proton efflux. The decrease in extracellular pH alters the activity of cell-wall-modifying proteins, including expansins, xyloglucan endoglycosylase/hydrolase (XTH), and PMEs [22,23,24], leading to changes in wall extension. Proton pumping also hyperpolarized PM, activating inward K^+^ channels and activating H^+^-coupled anion symporters (X^−^). This transport increases solute uptake activities and maintains the solute absorption required for water uptake and the pressure that forces the wall to expand.

The effect of auxin on cell expansion depends on both concentration and tissue. In general, auxin concentration can promote stem cell expansion and inhibit root cell expansion in physiology. We found that synthetic auxin inhibits root elongation. In addition, the auxin receptor antagonist auxin can restore the inhibitory effect of exogenous IAA on root growth. In addition, N-1-naphthalophthalic acid, a polar transport inhibitor of auxin, also affects root bending [25]. In studies on the auxin regulation of root cell expansion, apoplastic acidification induced by exogenous auxin can both promote and inhibit [26] root cell expansion and trigger apoplastic alkalization as well as acidification [27]. These paradoxical findings suggest that auxin plays a complex and dynamic role in controlling root cell pH homeostasis and cell expansion. Kinematic studies are needed to evaluate elongation beyond the meristem effect of auxin on root growth. Auxin-induced alkalization occurs within 15s [28,29]. Consistent with this extremely rapid alkalization is increased cytoplasmic Ca^2+^ ([Ca^2+^]_i_) levels. The increase in extracellular pH was inhibited both by pretreatment with the Ca^2+^ channel blocker La^3+^ [29,30] and by Ca^2+^ ionophores [30], suggesting that elevated [Ca^2+^]_i_ acts as a second messenger mediating auxin-induced alkalization.

Calcium ions (Ca^2+^) have been adopted as a ubiquitous intracellular second messenger, which plays an essential role in understanding a complicated network of plant growth and responses to abiotic and biotic stimuli, including salinity, cold, and drought [31,32]. Plant cells can rapidly change cytosolic free Ca^2+^ concentrations in a changing environment in time and space. The spatio-temporal patterning of cellular Ca^2+^ dynamics has been formulated as a concept of the Ca^2+^ signature. The Ca^2+^ signature, namely the alterations in amplitude, duration, frequency, and spatial distribution of the Ca^2+^ signal, encodes information about the type and the strength of the stimuli [33,34]. Downstream effectors decode such Ca^2+^-encoded stimulus-specific information. Most downstream effectors are Ca^2+^-sensing proteins, which bind to Ca^2+^ to initiate or regulate biochemical processes and ultimately translate information into specific end responses [35,36,37]. However, the mechanisms of how plant cells generate stimulus-specific Ca^2+^ signals have not yet been explored. There is a need to understand how plant cells initiate stimulus-specific Ca^2+^ signals and perceive and transduce signals to cope with unfavorable environmental conditions. Low pH stress is representative of such abiotic stress. However, the molecular mechanisms surrounding the initial perception of low pH stress are still unknown. *Arabidopsis* as a model plant was used to better understand the effects of low pH stress in cotyledons on basal Ca^2+^ signaling.

There are 24 h [Ca^2+^]_i_ oscillations of the cytosol and chloroplastic in light and dark cycles, or constant light, which rise to a peak of 300 nM, regulated by the circadian clock and light signaling [38,39,40,41,42]. Ca^2+^ oscillations of the cytosol are driven by the rhythmic production of cyclic ADP ribose [43] and are explicitly suppressed by the circadian oscillator gene CIRCADIAN CLOCK ASSOCIATED 1 (CCA1) [44]. Daily and circadian oscillations of Ca^2+^ regulate sense via a Ca^2+^ sensor protein CALMODULIN-LIKE 24, which interacts genetically with the circadian oscillator protein TIMING OF CAB 1 (TOC1) [45]. As aspects of photosynthesis, the changes in Ca^2+^ in the chloroplastic regulate the import of nuclear-encoded proteins and organelle division [46,47,48]. Additionally, the environmental transition between light and darkness produces a prolonged and sustained increase in Ca^2+^ that depends on the photoperiod [41,49,50].

This research simulated the normal plant growth environment in the image machine [51]. We also analyzed the relationship between low pH conditions and the basal Ca^2+^ concentration of *Arabidopsis* under a normal light/dark cycle. The main objective of this study was to investigate the effects of pH on changes in intracellular calcium levels and the plant’s cyclic response over a medium- to long-term period.

## 2. Materials and Methods

### 2.1. Plant Material and Growth Conditions

The *Arabidopsis thaliana* genotypes were used in the Col-0 background. Under the control of the Cauliflower Mosaic Virus 35S promoter, active aequorin (Ca^2+^ indicator) (pMAQ2) was obtained from Dr. Marc R. Knight [52]. Seeds were sterilized with 2.5% PPM (a plant preservative mixture; Caisson Laboratories), stratified at 4 °C for 3 days in the dark, and then transferred to a growth chamber. The growth chamber was configured with an LD light/dark cycle (16 h of light and 8 h of dark) at 22 ± 1 °C. *Arabidopsis* plants were grown in a 150 mm round Petri dish, which contained 1/2 Murashige and Skoog salts with 1.0% (*w*/*v*) sucrose (Sigma, St. Louis, MO, USA) and 1.2% (*w*/*v*) agar (Sigma) adjusted to pH 5.7 with KOH (Sigma).

### 2.2. Aequorin (AQ) Reconstitution and Plant Pretreatment

*Arabidopsis* plants expressing cytosolic apoaequorin were used for [Ca^2+^]_i_ measurements [52,53]. Seedlings were grown on a half-strength MS medium after 8 days and transferred onto a new half-strength MS medium in a 90 mm round Petri dish, which contains 1/2 Murashige and Skoog salts and 1.2% (*w*/*v*) agar without MES, adjusted to pH 5.8 with KOH. The culture medium was cut into a specific shape: a 5 cm striped strip and another 7 cm slope-type medium. The reconstitution of aequorin was performed in vivo by spraying seedlings with 0.8 mL of 50 μM coelenterazine (from Prolume) per Petri dish, followed by incubation at 22 °C in the dark for 8 h.

### 2.3. Low pH Treatment

Low-pH stress treatments were adopted from the experimental procedure described by [54]. For stress treatments, 4 Petri dishes were placed individually into the ChemiPro HT chamber. Two samples were repeated each time, and three independent experiments were performed. The treatment solution of 10 mM 2-Morpholinoethanesulfonic acid monohydrate (MES) (Sigma), which was adjusted to pH 5.0, 5.8, 6.0 with Tris, and pH 3.0, pH 4.0 with HCl, was added to a Petri dish in the dark. Aequorin luminescence was recorded for 20 min every 2 h after a switch from the light to the dark for 5 min as described [53,55]. The first 5 min imaging contained the autofluorescence of chlorophylls and was discarded. For Ca^2+^ channel blocking, seedlings were co-incubated with a treatment buffer containing 1 mM LaCl_3_. The total aequorin was estimated by discharging with 0.9 M CaCl2 in 10% (*v*/*v*) ethanol [55]. Experiments were carried out at room temperature (22–24 °C).

### 2.4. Measurement of Cytosolic Free Calcium ([Ca^2+^]_i_)

Treatments and aequorin luminescence imaging were performed at room temperature using a ChemiPro HT system [51], which includes a cryogenically cooled and back-illuminated charge-coupled device (CCD) camera, a liquid nitrogen autofiller, a camera controller, and computer-equipped WinView/32 software (Roper Scientific, Acton, MA, USA) [53]. The CCD camera has a 1300 × 1340-pixel resolution and was cooled to −120 °C by the cryogenic cooling system before image recording. The relative luminescence intensities were calculated as the ratio of aequorin luminescence intensity and discharged aequorin luminescence intensity [53,54,56].

### 2.5. Statistical Analysis

Microsoft Excel was used for data processing. A two-way analysis of variance (ANOVA) was implemented using SAS 9.1 software (SAS Institute, Cary, NC, USA). Means were separated using the least significant difference test at the 5% level. Values of *p* < 0.05 were considered statistically significant.

## 3. Result

### 3.1. pH-Induced Modulations in the [Ca^2+^]_i_ Oscillations Were Observed in Both the Leaves and Roots of Arabidopsis Seedlings

**Figure 1:**
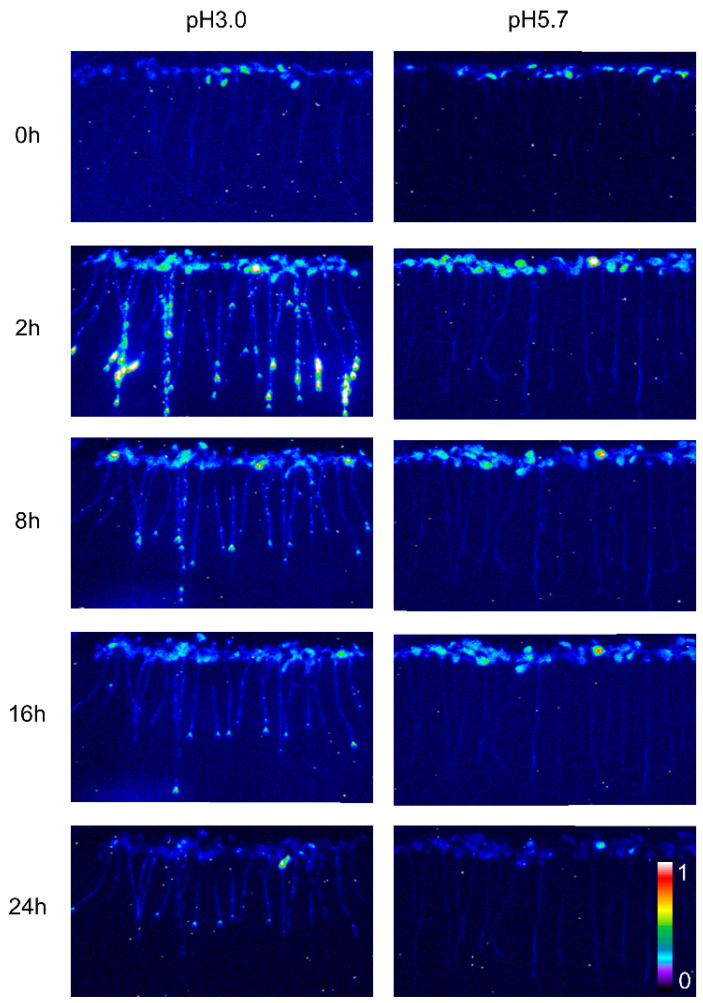
Spatio-temporal Ca^2+^ responses of *Arabidopsis* seedlings to low pH. The luminescence emitted by *Arabidopsis* seedlings expressing aequorin was captured using pseudocolored photon-counting images. In these images, cold colors (blue and green) denote regions with low luminescence counts, which correspond to low intracellular calcium ion concentrations ([Ca^2+^]_i_). Conversely, warm colors (yellow and orange) indicate regions of more intense luminescence, reflecting higher [Ca^2+^]_i_ levels. Prior to imaging, the seedlings were entrained to a 16 h light/8 h dark (16L/8D) photoperiod at an irradiance of approximately 90 μmol m^−2^ s^−1^ for a duration of 9 days. Sequential images were then acquired using a photon-counting camera, with integrations conducted every 20 min for a total of 24 h following exposure to low pH stimuli. The treatment solutions, consisting of a 1/2 MS medium without sugar, were adjusted to pH 3.0 (low pH) and pH 5.7 (control, CK) using a 1 M Tris base. Notably, only the roots of the *Arabidopsis* seedlings were subjected to these treatments, which were conducted on tilted media in each panel devoid of agar and EMS. During the treatment period, the photoperiod was maintained at 16L/8D, but each 1.5 h light and dark cycle was interrupted for 10 min for image acquisition, followed by a subsequent 20 min imaging session.

**Figure 2:**
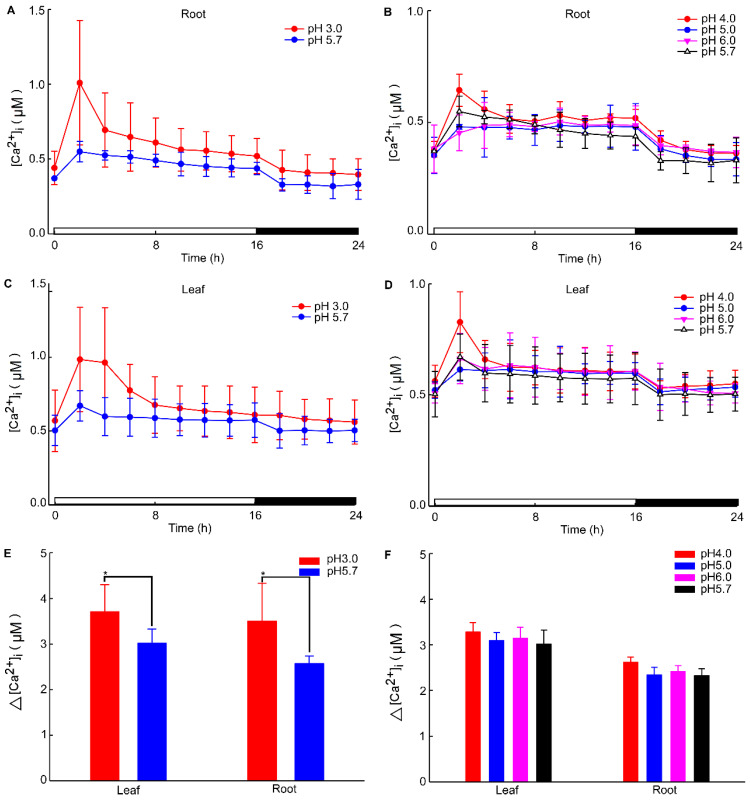
Increases in [Ca^2+^]_i_ in response to low pH treatments. Seedlings expressing aequorin and grown for a duration of 9 days were subjected to treatments with solutions whose pH levels were adjusted using a Tris base and EMS. Aequorin images were captured every 20 min within 2 h intervals for a total period of 24 h. The experiments aimed to investigate the increases in the intracellular calcium ion concentration ([Ca^2+^]_i_) induced by pH 3.0 and pH 5.7 treatments, specifically in the roots of *Arabidopsis* (**A**). Additionally, imaging was conducted to observe [Ca^2+^]_i_ increases in response to treatments with pH 4.0, pH 5.0, pH 5.7, and pH 6.0 solutions in the roots of *Arabidopsis* (**B**). Furthermore, this study explored the induction of [Ca^2+^]_i_ increases in *Arabidopsis* leaves by treating the roots with pH 3.0 and pH 5.7 solutions (**C**). Imaging was also performed to assess [Ca^2+^]_i_ increases in the leaves of *Arabidopsis* in response to treatments with pH 4.0, pH 5.0, pH 5.7, and pH 6.0 solutions applied to the roots (**D**). The overall changes in [Ca^2+^]_i_ in the roots and leaves of *Arabidopsis* under pH 3.0 and pH 5.7 conditions were analyzed (**E**). Similarly, the total change in [Ca^2+^]_i_ in the roots and leaves of *Arabidopsis* under pH 4.0, pH 5.0, pH 5.7, and pH 6.0 conditions was evaluated (**F**). The presented data represent the results of four independent experiments, with mean values and standard deviations (mean ± SD) calculated from a sample size of 180 (mean ± SD; n = 180).

**Figure A1:**
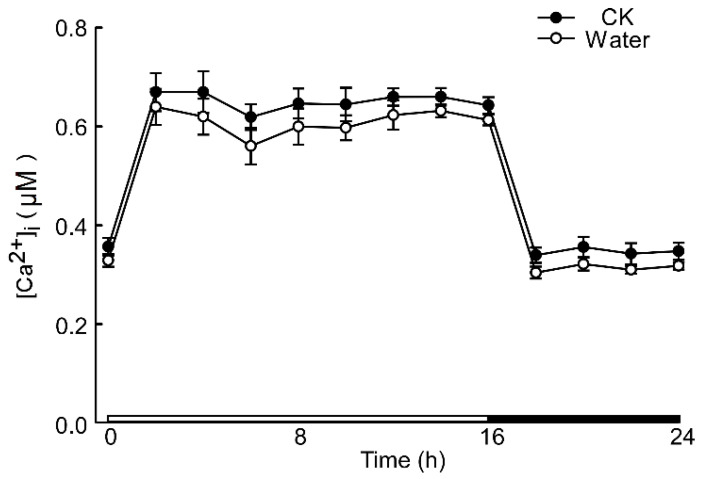
Increases in [Ca^2+^]_i_ in response to water. Aequorin-expressing seedlings grown for 9 days were treated with water and no treatment, and aequorin images were taken every 20 min for 2 h for 24 h—an increase in [Ca^2+^]_i_ induced by water and no treatment in root of *Arabidopsis* is shown. Data from four independent experiments are shown (mean ± s.e.m; n = 180).

The calcium signal in the root was significantly influenced by different levels of stress. Specifically, when *Arabidopsis* roots were subjected to acidic stress (pH 3.0), the calcium signal in both root and leaf tissues increased within 2–4 h under light conditions and subsequently remained at elevated levels compared to dark conditions (Figure 1). Notably, under acid stress, the calcium signal was observed to migrate towards the upper part of the root relative to the control group (pH 5.7) (Figure 1). However, for the circadian rhythm under the natural light cycle, *Arabidopsis* exhibited a periodic oscillation of calcium signals in its roots and leaves, both at pH 3.0 and in the control group. No significant differences in the Ca^2+^ signal or circadian rhythm were detected between *Arabidopsis* plants undergoing water treatment and those in the control group (Figure A1). During the same oscillation period, the amplitude of Ca^2+^ signals induced by various pH treatments differed, with low pH inducing a more pronounced Ca^2+^ signal. Additionally, the Ca^2+^ content in leaves was higher than in the roots, although the overall trend of the Ca^2+^ variation remained similar. At pH 3.0 and pH 4.0, *Arabidopsis* initially increased the Ca^2+^ signal, which then gradually decreased, indicating that the intracellular Ca^2+^ concentration returned to equilibrium (Figure 2A–D). After 4–8 h, the Ca^2+^ signals induced by pH 3.0 and pH 4.0 recovered to a static state, albeit with a higher baseline Ca^2+^ concentration compared to pH 5.0 and pH 6.0 (Figure 2A–D). Under natural light conditions, both pH 3.0 and the control group (pH 5.7) induced constant changes in [Ca^2+^]_i_ in the roots and leaves of *Arabidopsis* plants (Figure 2A,C). The leaf response to the Ca^2+^ signal recovered more slowly than the root response to acidic stimulation at pH 3.0 (Figure 2A,C). Nevertheless, the baseline [Ca^2+^]_i_ in *Arabidopsis* under light conditions was higher than under dark conditions (Figure 2A–D). The total change in the calcium signal over 24 h reflected the alteration in the Ca^2+^ content in the roots and leaves under a low acid stimulation, indicating significant changes in [Ca^2+^]_i_ in both leaves and roots at a pH of 3.0 (Figure 2E). Changes in the Ca^2+^ signal under pH 4.0 were less pronounced than those under pH 3.0 in the leaves while no significant difference was observed in the roots (Figure 2E,F).

### 3.2. pH Stimulation-Elicited [Ca^2+^]_i_ Oscillations in the Leaves and Roots of Arabidopsis Seedlings in the Presence of a Ca^2+^ Inhibitor

**Figure 3:**
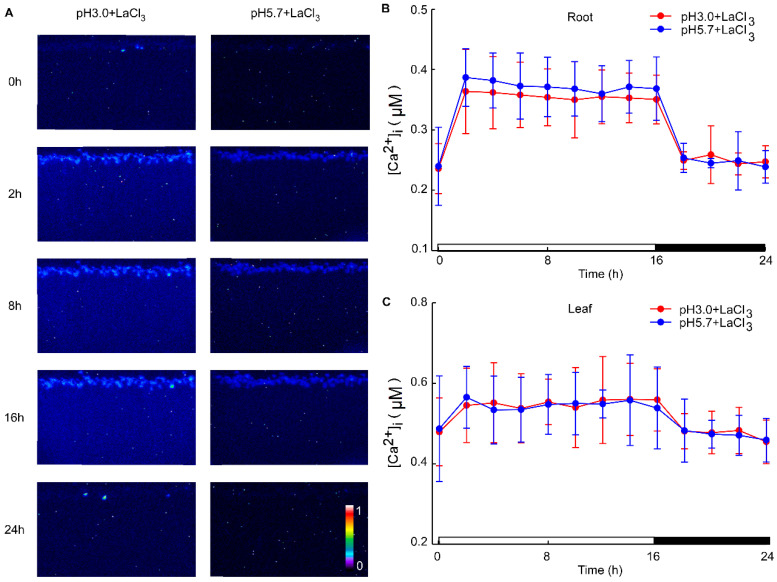
The effects of Ca^2+^ inhibitors on low pH-induced spatio-temporal [Ca^2+^]_i_ responses of Arabidopsis seedlings. The presented images show pseudocolored photons, counting the depictions of luminescence emitted by *Arabidopsis* seedlings expressing aequorin. These seedlings were entrained to a 16 h light/8 h dark (16L/8D) photoperiod at approximately 90 μmol m^−2^ s^−1^ for a duration of 9 days. Sequential images were captured using a photon-counting camera, employing a photon-counting integration interval of 20 min, with integrations conducted every 2 h over a 24 h period following exposure to low pH stimuli. The treatment solutions consisted of pH 3.0 (representing low pH) and pH 5.7 (serving as the control, denoted as CK), both containing 1 mM Ca^2+^ inhibitor (LaCl_3_), adjusted with a 1 M Tris base. Notably, these solutions were specifically applied to the roots of *Arabidopsis* seedlings, which were placed on a tilted medium in each panel devoid of agar and EMS. During the treatment phase, the photoperiod was maintained at 16L/8D, but with each 1.5 h light and dark cycle processed for 10 min, followed by imaging for 20 min (**A**). The administration of Ca^2+^ inhibitors (1 mM LaCl_3_) was observed to reduce pH 3.0 and pH 5.7-induced changes in the intracellular Ca^2+^ concentration ([Ca^2+^]_i_) in the roots of *Arabidopsis* (**B**). Similarly, treatment with Ca^2+^ inhibitors (1 mM LaCl_3_) mitigated [Ca^2+^]_i_ changes in the leaves of *Arabidopsis*, which were elicited by exposure to pH 3.0 and pH 5.7 solutions applied to the roots of the seedlings (**C**). The data presented are derived from four independent experiments (mean ± SD; n = 180).

Under acidic stress conditions, the leaves exhibit the ability to sense acid stimulation originating from the roots. To investigate this phenomenon, we monitored alterations in calcium signals within the leaf while inhibiting the root with LaCl_3_ under acid stimulation. The La^3+^ ion, previously established as an agonist of Ca^2+^ channel blockers, was utilized to inhibit Ca^2+^ influx [57]. Despite the presence of the 1mM LaCl_3_ inhibitor, variations in pH did not alter the levels of Ca^2+^ in the leaves and roots of *Arabidopsis*. Notably, specific concentrations of LaCl_3_ demonstrated significant inhibitory effects on the low pH-triggered increase in [Ca^2+^]_i_ (Figure A3). When comparing images at pH 3.0 and pH 5.7, no discernible differences in Ca^2+^ levels between the roots and leaves of *Arabidopsis* were observed, although variations in Ca^2+^ levels due to the photoperiod were present (Figure 3A). Across different pH treatments, there were no substantial changes in Ca^2+^ levels detected in the roots and leaves of *Arabidopsis* (Figure 3B,C, Figure A4A–D and Figure A5A–D). Distinct from the observations in Figure 1 and Figure 2, the pH did not significantly elicit alterations in Ca^2+^ levels in *Arabidopsis* when LaCl_3_ inhibition was present.

**Figure 4:**
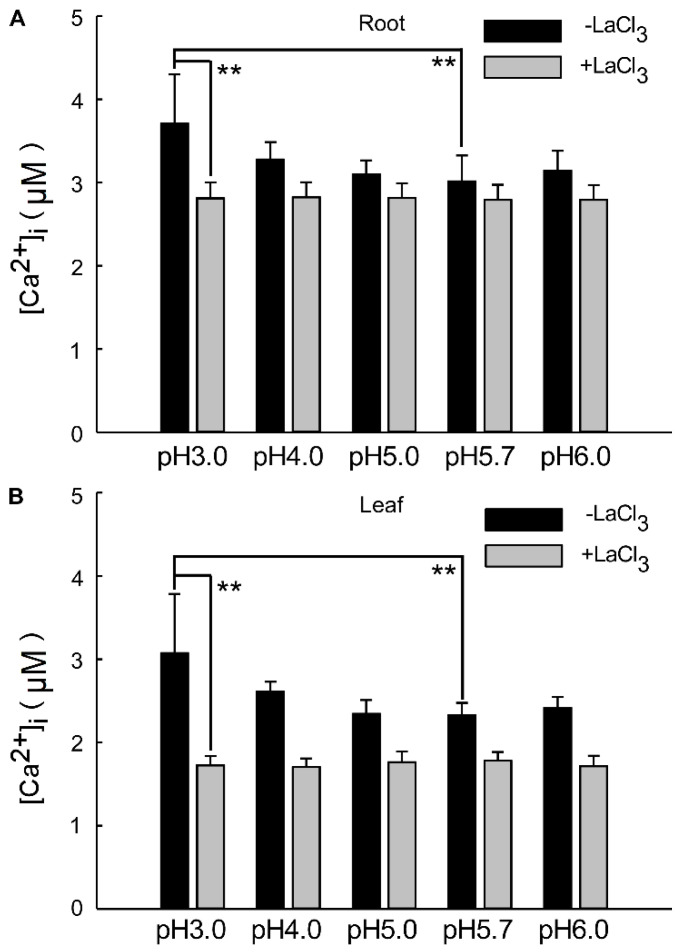
Changes in [Ca^2+^]_i_ in *Arabidopsis* under low pH in the presence of Ca^2+^ inhibitors. Under various pH conditions, specifically pH 3.0, pH 4.0, pH 5.0, pH 5.7, and pH 6.0 (as depicted in Figure 2 and Figure 4), the quantity of the intracellular Ca^2+^ concentration ([Ca^2+^]_i_) was elicited by acid stimulation in the roots of *Arabidopsis* exhibited alterations. However, in the presence of inhibitors, the [Ca^2+^]_i_ levels in the roots remained unaltered (**A**). Similarly, under the aforementioned pH conditions (Figure 2 and Figure 4), the magnitude of [Ca^2+^]_i_ in the leaves of *Arabidopsis*, which was induced by acid stimulation in the roots, also demonstrated changes. Nonetheless, when inhibitors were present, the [Ca^2+^]_i_ levels in the leaves remained unchanged (**B**).

The overall change in calcium signals over a 24 h period reflects the alteration in Ca^2+^ content within the roots and leaves under a low-acid stimulation. This suggests that significant changes in [Ca^2+^]_i_ occur in the leaves and roots of the plant, specifically under pH 3.0 conditions compared to the control. The alteration in Ca^2+^ signals under pH 4.0 was less pronounced than under pH 3.0 in both the leaf and root (Figure 4A,B). As depicted in Figure 1 and Figure 3, seedlings exhibited varying responses to different acidic treatments. Acid stimuli significantly induced Ca^2+^ signals, with notable differences in acidic treatments and signal amplitudes between the shoot and root. The amplitude of Ca^2+^ signals was significantly enhanced under pH 3.0, correlating with the intensity of the H^+^ stimulus and persisting for an extended period across all low pH conditions. The amplitudes of [Ca^2+^]_i_ induced by the low pH 3.0 in *Arabidopsis* roots were 0.36 times higher than those induced by pH 5.7. However, under LaCl_3_ inhibition, the distinction between them was insignificant across all acidic treatments (Figure 4A). Similarly, the pH 3.0-induced [Ca^2+^]_i_ amplitude was 0.51-fold higher than pH 5.7 in the leaves, but this difference became negligible under LaCl_3_ inhibition (Figure 4B).

### 3.3. Prolonged Exposure to pH Stimulation-Induced [Ca^2+^]_i_ Oscillations in the Leaves of Arabidopsis Seedlings

**Figure 5:**
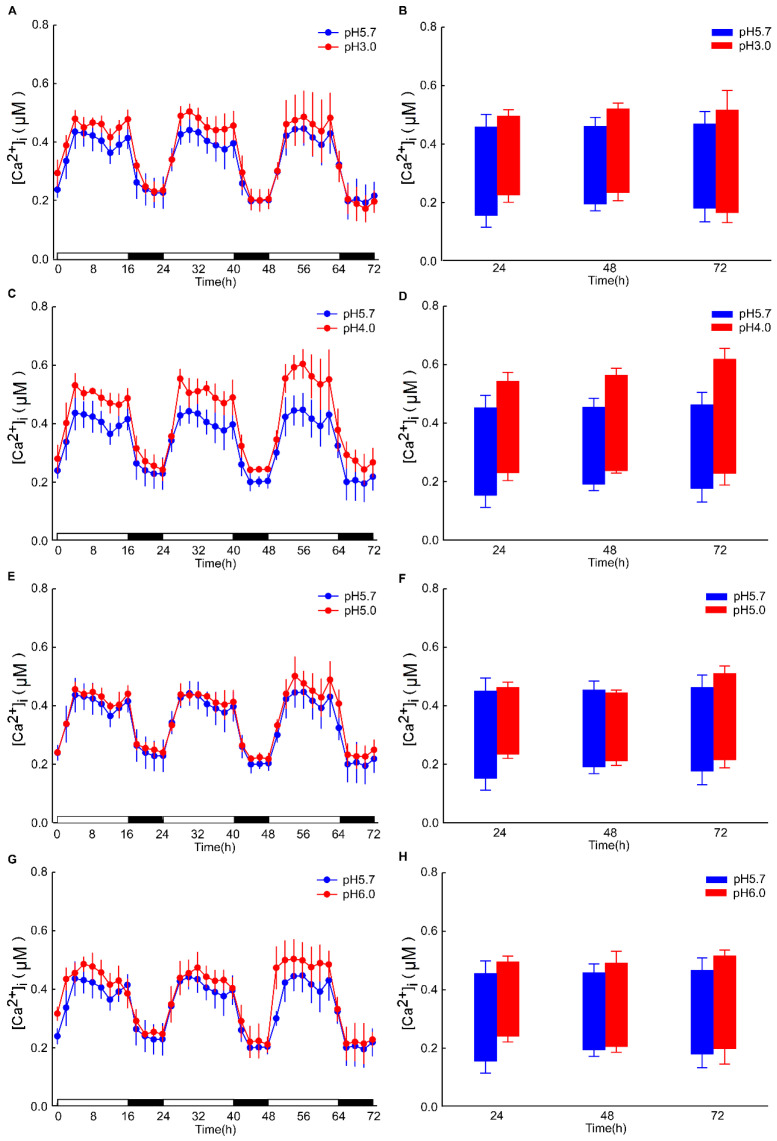
Low pH modulates the amplitude of oscillations in [Ca^2+^]_i_. seedlings, which were entrained to a 16L/8D photoperiod at an irradiance of approximately 90 μmol m^−2^ s^−1^ for a duration of 9 days. Sequential images were captured utilizing a photon-counting camera, with a photon-counting integration interval set at 20 min. These integrations were performed every 2 h following low pH stimuli, which were applied 8 h into the dark period. In comparison to the pH 5.7 condition, notable differences were observed in the amplitude of [Ca^2+^]_i_ oscillations elicited by the pH 3.0 (**A**), pH 4.0 (**C**), pH 5.0 (**E**), and pH 6.0 (**G**) stimulation in the leaves of *Arabidopsis*. The treatment solution consisted of a 1/2 MS medium without sugar, adjusted using a 1 M Tris base, and was applied solely to the roots of *Arabidopsis* seedlings placed on tilted media in each panel, devoid of agar and EMS. During the treatment phase, the photoperiod remained constant at 16L/8D, but each 1.5 h light and dark cycle was followed by a 10 min processing period, subsequent to which imaging was conducted for 20 min. A box plot is presented, illustrating the upper and lower limits of the primary [Ca^2+^]_i_ oscillations induced by pH 3.0 (**B**), pH 4.0 (**D**), pH 5.0 (**F**), and pH 6.0 (**H**) stimulations in the leaves of *Arabidopsis*, relative to the pH 5.7 condition. The data presented are derived from four independent experiments (mean ± s.e.m; n = 180).

**Figure A2:**
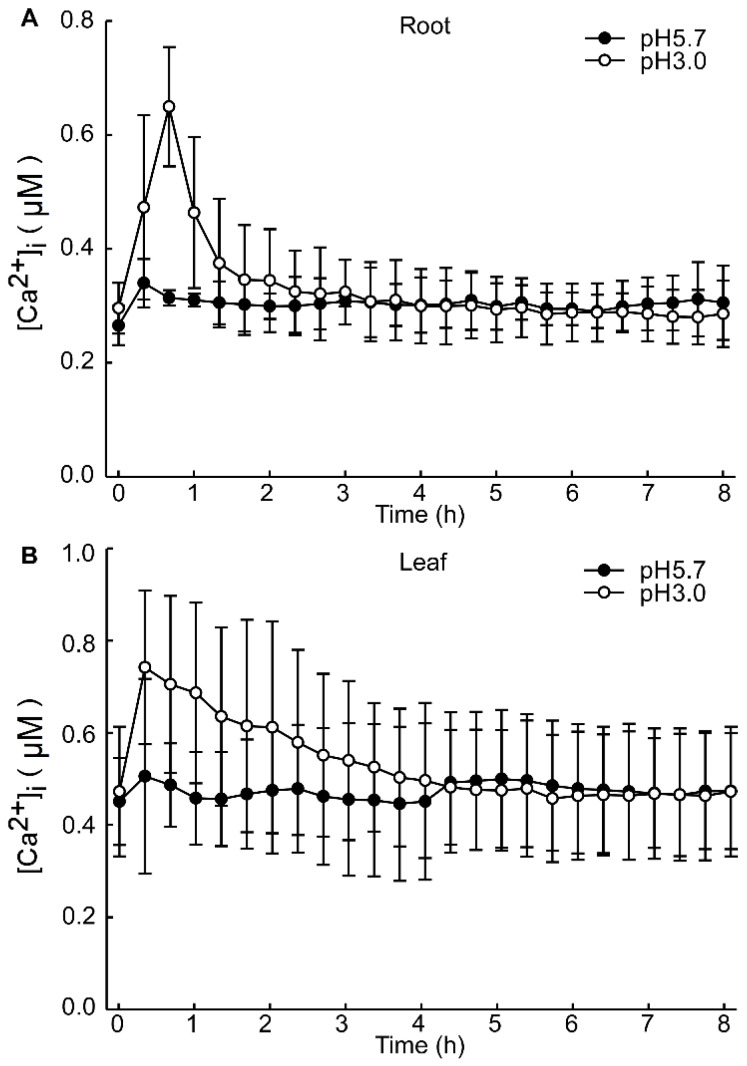
Increases in [Ca^2+^]_i_ in response to low pH after 8 h. Aequorin-expressing seedlings grown for 9 days were treated with solutions containing EMS-adjusted pH with Tris base, and aequorin images were taken every 20 min for 8 h. Increase in [Ca^2+^]_i_ induced by pH 3.0 and pH 5.7 treatment in root of *Arabidopsis* (**A**). Increase in [Ca^2+^]_i_ in *Arabidopsis* leaf, induced by pH 3.0 and pH 5.7 solution treated on root of *Arabidopsis* (**B**). Data from four independent experiments are shown (mean ± s.e.m; n = 180).

The intracellular Ca^2+^ ([Ca^2+^]_i_) changes in *Arabidopsis* after 4 h exhibited minimal significance under dark conditions. However, the alterations in the basal intracellular Ca^2+^ levels in *Arabidopsis*, induced by different pH conditions, are discernible (Figure A2). Specifically, under pH 3.0, the response to the Ca^2+^ signal is rapidly augmented in the leaf, whereas the response is retarded in the root. These results are analogous to those observed under light/dark (LD) conditions (Figure 2A,C). After 8 h of acid stimulation in the dark condition, the intracellular calcium signal resonance gradually stabilized. Nevertheless, under light conditions, pH 3.0 and 4.0 lead to an increase in Ca^2+^ levels in the leaves of *Arabidopsis*. Under prolonged conditions, at pH 4.0, the basal [Ca^2+^]_i_ level of *Arabidopsis* over 72 h was sustained at a higher level relative to pH 3.0 (Figure 5A,C). Conversely, the basal [Ca^2+^]_i_ level remained comparable to other pH conditions (Figure 5E,G). Notably, under pH 4.0, the amplitude of the Ca^2+^ signal was more pronounced than under other conditions. In contrast, under the remaining pH conditions, the amplitude of the Ca^2+^ signal remained unchanged (Figure 5B,D).

## 4. Discussion

Previous research on pH and calcium ions has primarily focused on the correlation of Ca^2+^ signals to the biological clock, indicating periodic oscillations of [Ca^2+^]_i_ [40,58]. In this study, under the acid stimulation of pH 3.5 and 4.5, the Ca^2+^ signal was observed to be delayed. However, the more potent acid stimulation (pH 3.5) elicited a more evident response than the acid stimulation at pH 4.5, with a more considerable increase in Ca^2+^ levels initiated within a few minutes [59]. Building upon these two methods, we have refined the experimental approach, enabling us to use AQ to study the response of pH to the long-term calcium ion dynamics in the plant *Arabidopsis*. In this paper, we specifically focus on responses and variations in [Ca^2+^]_i_ in *Arabidopsis* over an extended period. Given that the nature of the medium determines the limitations of the low pH conditions and the pH properties, we adopted a unique pH system that influences [Ca^2+^]_i_ changes in *Arabidopsis* [51]. This system allowed us to study the changes in the [Ca^2+^]_i_ under low pH stress over a prolonged duration. In our experiment, an overdose of coelenterazine was administered. We employed a recording system to compare various Ca^2+^ responses under a low pH in *Arabidopsis* seedlings. The bioluminescence emitted by each seedling cluster was recorded for 1200 s, every 2 h, over a 24 h period. The Ca^2+^ recordings revealed tissue-specific Ca^2+^ signals in the shoot and root associated with low pH acidic stress.

Calcium ions (Ca^2+^) have been recognized as a ubiquitous second messenger, playing a pivotal role in plant signal transduction [32,60]. Aequorin is an early version of the Ca^2+^ indicator, with the intensity of its bioluminescence contributing to the elucidation of the Ca^2+^ signature in plants [52]. The transformation of aequorin in plants has proven to be a valuable tool for the non-invasive investigation of [Ca^2+^]_i_ signatures. It is known that specific stimuli, including abiotic and biotic stresses, hormones, and amino acids, can elicit unique spatio-temporal patterns of Ca^2+^ signatures in most aspects of whole seedlings [59,61,62,63]. The basal [Ca^2+^]_i_ is maintained at a concentration of approximately 100 nM, which is about 20,000-fold lower than the extracellular Ca^2+^ concentration [32,64,65]. The stimulus may rapidly increase [Ca^2+^]_i_ spikes or induce complex [Ca^2+^]_i_ oscillations recurring within a period of 1 to 20 min [66,67,68]. However, long-term [Ca^2+^]_i_ oscillations, characterized by a 24 h period, have been observed in the cytosol and chloroplast of plants, as well as in the cytosol of the suprachiasmatic nucleus of the mouse [40,69].

We conducted an investigation into the basal [Ca^2+^]_i_ response to varying pH levels. Specifically, we observed a slow kurtosis in [Ca^2+^]_i_ increases in response to pH 3.0 compared to pH 5.7 during the first 8 h (Figure 1 and Figure 2A). Notably, the peak [Ca^2+^]_i_ increased when induced by pH 4.0 and was less pronounced than that elicited by pH 3.0 (Figure 2A). Moreover, other pH values (pH 5.0, pH 5.7, pH 6.0) did not significantly increase [Ca^2+^]_i_ compared to the acidic stress induced by pH 3.0 and pH 4.0 (Figure 2A,B). To our knowledge, such a systematic comparison, which is essential for understanding Aequorin-recorded Ca^2+^ signals, has not been previously conducted in *Arabidopsis*. This recording system can serve as a valuable tool for identifying potentially novel components of the Ca^2+^ signal in response to acidic stress. Although only *Arabidopsis* roots were directly stimulated by acid stress, which led to an increase in Ca^2+^, the stimulation of the leaves also resulted in an increase in intracellular Ca^2+^, albeit with a prolonged response rate. These data are consistent with the transmission of calcium signals stimulated by other stresses [70], indicating that acid stress can increase [Ca^2+^]_i_ signaling and that the [Ca^2+^]_i_ signal can also be transmitted upwards as a systemic wound signal [71,72].

Auxin stimulates growth and exerts an influence on the PM H^+^-ATP complex, sustaining protons pumping phosphorylation and ultimately leading to proton efflux, potentially through apoplast acidification. Treatments with acid buffers have been observed to stimulate hypocotyl segment elongation [73,74,75]. The growth response is immediate, with a decrease in pH preceding growth but occurring after auxin treatment [76,77]. In response to acid buffer stimulation at pH 3.5 and pH 4.5, a Ca^2+^ signal is triggered within a few minutes. However, the more potent acid stimulation at pH 3.5 is initiated earlier, and the Ca^2+^ increase is more pronounced than that elicited by the acidic stimulus at pH 4.5 [59]. In this research, changes in [Ca^2+^]_i_ were observed in the seedlings under acid stimulation during a light/dark (LD) cycle. Since light also causes an increase in calcium levels in plants during the LD growth cycle, the [Ca^2+^]_i_ levels in plants decrease to lower levels in darkness [38,45]. When plants are transferred to dark conditions, the [Ca^2+^]_i_ in the plant seedlings still maintain this periodic [Ca^2+^]_i_ oscillation cycle [45,48]. Therefore, our results presented in Figure 2 demonstrate that the oscillation of [Ca^2+^]_i_ is not only included by light but also induced by acid stress.

**Figure A3:**
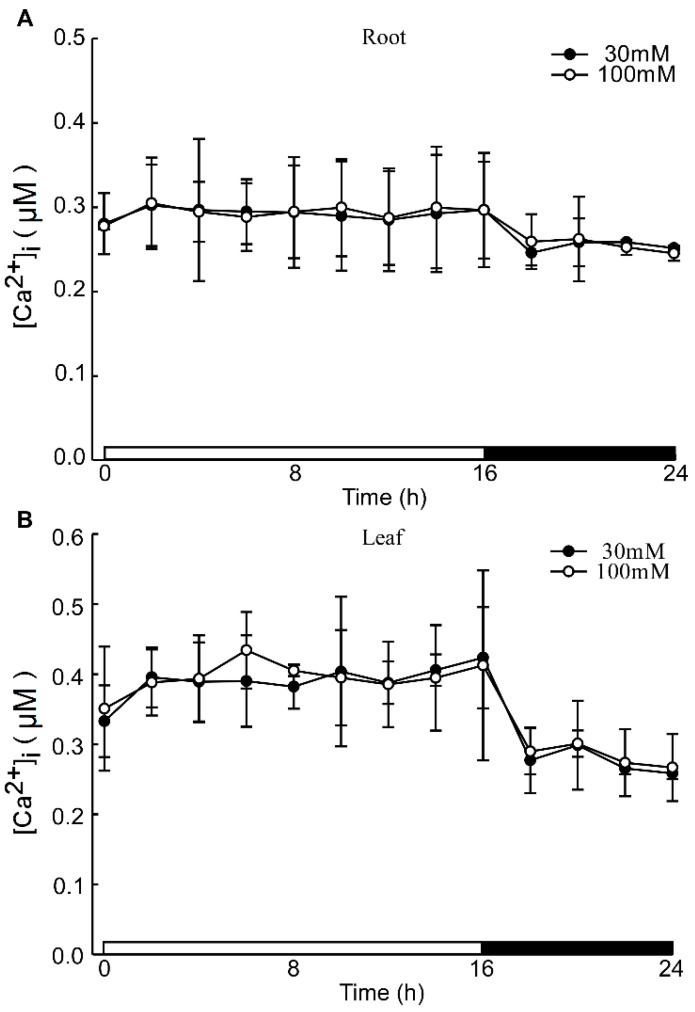
The effects of Ca^2+^ inhibitors on low pH-induced [Ca^2+^]_i_ increases. The effects of different concentrations of Ca^2+^ inhibitors in pH 3.0 induced [Ca^2+^]_i_ increases. The concentration gradient of LaCl_3_ was 30 μM and 100 μM. Data from four independent experiments are shown (mean ± sd; n = 10; NS, insignificant *p* > 0.05; Student’s *t*-test).

**Figure A4:**
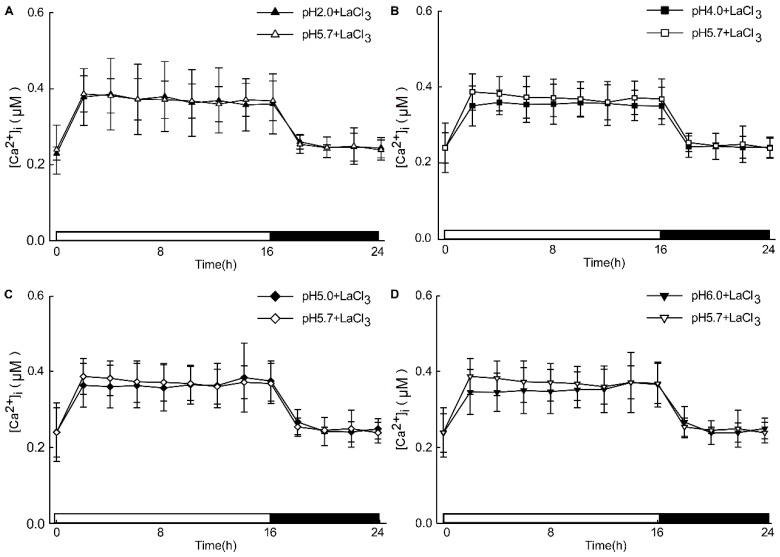
The effects of Ca^2+^ inhibitors on low pH-induced [Ca^2+^]_i_ increases in the root. Seedlings expressing aequorin and grown for 9 days were treated with solutions containing 1mM LaCl_3_ with an adjusted pH with Tris base, and aequorin images were taken every 20 min in 2 h for 24 h. The treatment of Ca^2+^ inhibitors (1 mM of LaCl_3_) reduced pH 2.0, and pH 5.7 induced [Ca^2+^]_i_ changes in the *Arabidopsis* root (**A**). The treatment of Ca^2+^ inhibitors (1 mM of LaCl_3_) reduced [Ca^2+^]_i_ changes in the leaves of *Arabidopsis*, which were induced by pH 4.0 and treated with a pH 5.7 solution (**B**). The treatment of Ca^2+^ inhibitors (1 mM of LaCl_3_) reduced [Ca^2+^]_i_ changes in the leaves of *Arabidopsis*, which were induced by pH 5.0 and treated with a pH 5.7 solution (**C**). The treatment of Ca^2+^ inhibitors (1 mM of LaCl_3_) reduced [Ca^2+^]_i_ changes in the leaves of *Arabidopsis*, which were induced by pH 6.0 and treated with a pH 5.7 solution (**D**). Data from four independent experiments are shown (mean ± SD; n = 180).

**Figure A5:**
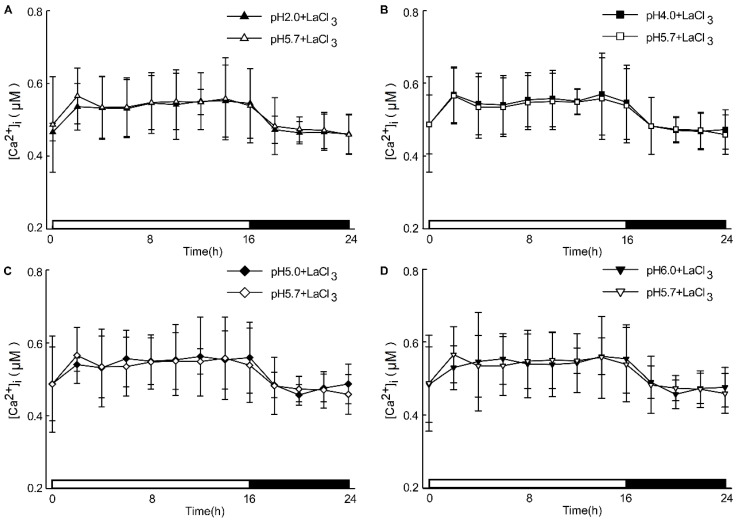
The effects of Ca^2+^ inhibitors on low pH-induced [Ca^2+^]_i_ increases in leaves. Seedlings expressing aequorin and grown for 9 days were treated with solutions containing 1 mM LaCl_3_ with an adjusted pH with Tris base, and aequorin images were taken every 20 min in 2 h for 24 h. The treatment of Ca^2+^ inhibitors (1 mM of LaCl_3_) reduced pH 2.0, and pH 5.7 induced [Ca^2+^]_i_ changes in *Arabidopsis* leaves (**A**). The treatment of Ca^2+^ inhibitors (1 mM of LaCl_3_) reduced [Ca^2+^]_i_ changes in the leaves of *Arabidopsis*, which were induced by pH 4.0 and treated with a pH 5.7 solution (**B**). The treatment of Ca^2+^ inhibitors (1 mM of LaCl_3_) reduced [Ca^2+^]_i_ changes in the leaves of *Arabidopsis*, which were induced by pH 5.0 and treated with a pH 5.7 solution (**C**). The treatment of Ca^2+^ inhibitors (1 mM of LaCl_3_) reduced [Ca^2+^]_i_ changes in *Arabidopsis* leaves, which was induced by pH 6.0 and treated with a pH 5.7 solution (**D**). Data from four independent experiments are shown (mean ± SD; n = 180).

The increase in extracellular pH was inhibited by pretreatment with the Ca^2+^ channel blocker La^3+^ [29,30]. The La^3+^ ion has been used as an agonist of Ca^2+^ channel blockers to suppress Ca^2+^ influx [57,78]. No significant differences were observed between concentrations of 0.03 mM, 0.1 mM, and 1 mM, indicating that LaCl_3_ effectively blocked the saturation of Ca^2+^ channels (Figure A3). Specifically, 1 mM LaCl_3_ completely inhibited the increase in [Ca^2+^]_i_ in response to acidic stress for 24 h. In the presence of LaCl_3_, after 1.5 h, the [Ca^2+^]_i_ levels in *Arabidopsis* did not change across different acidic pH conditions, suggesting that LaCl_3_ could effectively inhibit the influx of [Ca^2+^]_i_ in plants under acid stress (Figure 1 and Figure 3A). Although acid stress-induced changes in [Ca^2+^]_i_ in plants were abrogated, inherent light-induced [Ca^2+^]_i_ signals are still detectable (Figure 3B,C). These findings imply that the influx of extracellular Ca^2+^ primarily mediates the Ca^2+^ signal generated by acid stimulation in the roots. When Ca^2+^ channels are blocked, Ca^2+^ may not enter cells to form calcium signals and cannot be transmitted upward (Figure A3). Concurrently, due to the incomplete inhibition of H^+^ channels, excessive H^+^ could still enter cells. However, while the intracellular pH of root cells may change, the intracellular Ca^2+^ signal does not increase, and Ca^2+^ signals are not transmitted upward to the leaves. This may be attributed to the intracellular calcium store not releasing Ca^2+^ into the cytoplasm to form a detectable calcium signal response to low pH.

The activation of apoplast acidification occurs through the hyperpolarization of PM H^+^-ATPase, ultimately leading to cell wall loosening, solute and water uptake, elevated turgor pressure, and cell expansion [79]. α- and β-expansions are activated at a low pH of approximately 4.0, playing a crucial role in regulating the structure and relaxation of the cellulose–xyloglucan network within the cell walls [80]. The low pH environment modulates the stability of the PME3-PMEI7 complex and inhibits PME3 activity, although other PME complex formations may exhibit pH insensitivity [81]. Short galacturonic acid (GalA) fragments, specifically GalA3-4, have been found to stimulate hypocotyl growth in dark conditions, suggesting that specific cell wall components are involved in the cell-to-cell communications linked to expansion [82]. In this research, stimulation with pH 3.0 on the root resulted in an increase in [Ca^2+^]_i_ in both the leaves and roots of *Arabidopsis*, particularly within the first 4 h under dark conditions. At pH 3.0, the leaf response to the Ca^2+^ signal was rapidly augmented, albeit with a slight response compared to the roots. These findings align with observations made under LD light conditions (Figure 2A,C). Notably, the changes in intracellular [Ca^2+^]_i_ changes in the *Arabidopsis* leaf after 4 h were insignificant under dark conditions (Figure A2). These results imply that a low pH may exert an influence on cell wall structures and biophysical properties, which constitute a complex network orchestrating polar cell growth.

Under both light and dark conditions, the plant exhibits a prolonged and sustained increase in Ca^2+^ levels, which is dependent on the photoperiod [41,49,50]. This increase is driven by cyclic adenosine diphosphate ribose-mediated Ca^2+^ release from internal stores [44,83], serving to encode information regarding photoperiod, timing, and light intensity [40,84]. Tonoplast-localized Ca^2+^/H^+^ antiporters, such as CAX, elevate Ca^2+^ concentration in the apoplastic, which is concomitant with altered cell wall mechanical properties, the reduced expression of cell wall-modifying agent transcripts, an increase in apoplastic pH, and perturbed auxin transport [85,86]. Additionally, the vacuolar H+–pyrophosphatase AVP1 has been implicated in alterations in auxin efflux and changes in apoplastic pH [87,88]. Despite these findings, the specific functions regulated by circadian oscillations of [Ca^2+^]_i_ remain unidentified. In this research, intracellular calcium signals were observed to gradually stabilize after 8 h of acid stimulation under dark conditions. We investigated the changes in intracellular calcium in *Arabidopsis* following 8 h of acid stimulation, particularly focusing on the alterations in basal [Ca^2+^]_I_ levels. Our comparisons were made across pH levels of 5.0, 5.7, and 6.0, where no discernible differences in intracellular calcium in *Arabidopsis* were recorded. However, under pH 4.0 conditions, the level of intracellular basal calcium in *Arabidopsis* was significantly higher than that observed under pH 3.0 conditions. This result indicates that acid stimulation can continuously influence plants’ intracellular [Ca^2+^]_i_ under light conditions and also that suitable low pH conditions may affect basal [Ca^2+^]_i_ levels, as well as the growth and development of plants. Our study demonstrated that low pH stress triggers the [Ca^2+^]_i_ signaling of calcium oscillation in the *Arabidopsis* cytoplasm, along with the [Ca^2+^]_i_ influx from the apoplastic to the cytoplasm, independent of the [Ca^2+^]_i_ efflux from calcium stored in the vacuole. It is plausible that a suitable low pH of 4.0 may serve as a spatial-temporal input into the circadian clock system.

## 5. Conclusions

In conclusion, our findings reveal that *Arabidopsis* roots exhibit variations in basal Ca^2+^ levels when subjected to low pH stress, with a notable increase observed in response to decreasing pH values. This elevation in basal Ca^2+^ levels was mirrored in the leaves. Interestingly, the induction of basal Ca^2+^ levels by a low pH exceeded normal levels under light conditions but returned to the baseline under dark conditions. Furthermore, the presence of La^3+^ inhibited extracellular Ca^2+^ influx, preventing significant increases in basal Ca^2+^ levels in both the roots and leaves. This suggests that H^+^ is unable to trigger Ca^2+^ efflux from the vacuoles to the cytoplasm, thereby sustaining relatively high basal Ca^2+^ levels. Notably, under the conditions of an extended photoperiod, the elevated basal Ca^2+^ levels in leaves, which were initially induced in the roots, remained consistently high when exposed to a specific pH stress. The benefits of these results for researchers are significant as they provide new insights into the mechanisms underlying plant responses to low pH stress, particularly in terms of calcium signaling. These findings open up new avenues for further research into the role of calcium signaling in plant responses to environmental stresses, such as low pH. The discovery that extended photoperiods maintain high basal Ca^2+^ levels in leaves under specific pH stress suggests a potential link between light signaling and calcium homeostasis, which warrants further investigation. Additionally, the study’s findings may have implications for crop improvement and stress tolerance, as understanding the mechanisms of calcium signaling in response to low pH stress could lead to the development of more resilient plant varieties.

## Data Availability

Data are contained within the article.

## Abbreviations

[Ca^2+^]_i_, cytosolic free Ca^2+^ concentration; AQ, aequorin.
